# Effects of microbial inoculant and additives on pile composting of cow manure

**DOI:** 10.3389/fmicb.2022.1084171

**Published:** 2023-01-05

**Authors:** Qian Yang, Shiqiu Zhang, Xueping Li, Kun Rong, Jialiang Li, Lihua Jiang

**Affiliations:** ^1^College of Biological and Environmental Engineering, Binzhou University, Binzhou, China; ^2^Key Laboratory of Eco-Environmental Science for Yellow River Delta, Binzhou University, Binzhou, China; ^3^College of Chemistry, Chemical Engineering and Materials Science, Shandong Normal University, Jinan, China; ^4^College of Resources and Environmental Engineering, Shandong Agricultural and Engineering University, Jinan, China; ^5^Binzhou Jingyang Biological Fertilizer Co., Ltd., Binzhou, China

**Keywords:** cow manure, aerobic composting, microbial inoculant, organic fertilizer additives, pile composting

## Abstract

Composting is an effective method of recycling organic solid waste, and it is the key process linking planting with recycling. To explore the reuse of agricultural organic solid waste as a resource in the Yellow River Delta, the effects of microbial inoculant and different additives (calcium superphosphate, biochar, tomato straw, rice husk, and sugar residue) on pile composting of cow dung were studied to obtain the best composting conditions. The results showed that microbial inoculant and additives all played positive roles in the process of aerobic composting, and the experimental groups outperformed the control groups without any additives. For discussion, the microbial inoculant promoted rapid pile body heating more than the recovery materials alone, and the effects on aerobic composting were related to the organic matter of substrates and biochar. After being composted, all the materials were satisfactorily decomposed. Degradation of additives into humic acid might serve as electron shuttles to promote thorough organic matter decomposition. These results provide a scientific basis data for industrial composting of organic solid waste processed by on-site stacking, and provide a reference for researcher and practitioners for studying the applications of microbial inoculant on aerobic composting.

## Graphical Abstract

Effects of microbial inoculant and additives on pile co-composting. 
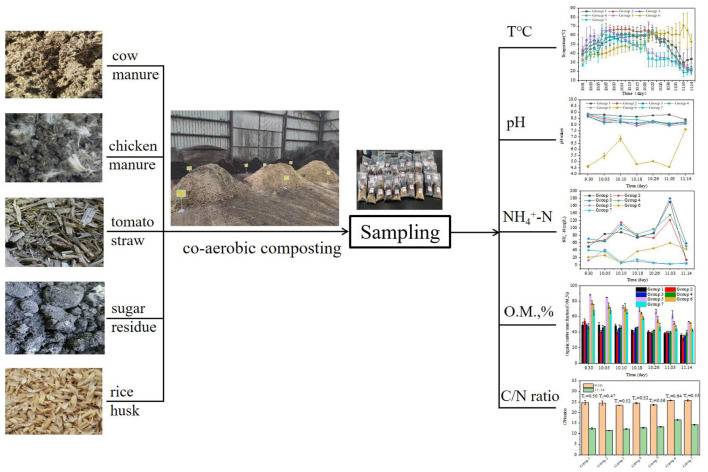

## Introduction

Animal manure is valuable renewable resource due to having the abundant organic matter (Liu et al., 2021; Fang et al., 2022). The annual production of livestock manure only in China has reached 3.8 billion tons (Guo et al., 2020). After General Secretary Xi Jinping inspected the Yellow River estuary in Dongying, Shandong Province, China in October 2021, he initiated the first steps of ecological protection and high-quality development in the Yellow River Basin. As an important part of the Yellow River Basin, the Yellow River Delta is rich in land resources, but the soil is salinized to various degrees. Yangxin County, Binzhou City, Shandong Province, which is located in the Yellow River Delta development zone, is one of the first pilot counties for comprehensive land use reform in China. It is one of the top 100 animal husbandry counties, mainly through its raising of cattle. The county produces huge amounts of livestock and poultry manure and crop straw each year, and there is currently no effective treatment method. In order to strengthen the ecology and environment of the Yellow River Delta, improve soil fertility, and achieve high-quality development of agriculture in the basin, exploring the combination of resource utilization of organic solid waste and soil fertility improvement in this region has become a critical research focus.

Composting is an effective way to recycle organic solid waste, bringing together crop planting and recycling (Briški et al., 2003; Liao et al., 2019; Hu et al., 2022). To improve soil fertility in saline-alkali land, the application of organic fertilizer is particularly effective (Orden et al., 2022; Zou et al., 2022). Aerobic composting is an effective way to realize the reduction, reuse, and harmless treatment of organic waste. However, traditional aerobic composting has problems such as its long fermentation period, slow degradation of organic matter, low degree of humification, difficulty in decomposing some materials, and safe application of compost products, which have become the key factors restricting the industrialization of aerobic composting (Chen et al., 2015; Wang and Zeng, 2018). In order to improve the fermentation speed and reduce the loss of carbon and nitrogen, various additives, such as crop straw, biochar, calcium superphosphate, and even microbial inoculant have been often added to compost (Li M. et al., 2008; Zeng et al., 2008; Huang et al., 2013; Wu et al., 2017; He et al., 2018; Sanchez-Monedero et al., 2018; Zhou et al., 2019; Guo et al., 2021; Mei et al., 2022).

Additives can be divided into organic additives, inorganic additives, and microorganisms according to their sources (Barthod et al., 2018). There are many kinds of inorganic additives (Barrington et al., 2002; Yang et al., 2013; Wang et al., 2016). They are characterized by porosity and high specific surface area (which is conducive to the growth and reproduction of microorganisms), can effectively improve compost compaction and ventilation, retain compost nutrients, slow the release of compost gases, strengthen the retention of carbon and nitrogen elements, and reduce the ecological toxicity of heavy metals, antibiotics, and other pollutants in compost (Wang and Zeng, 2018; Akdeniz, 2019). With the recent advocacy for a dual-carbon target (Chen and Liu, 2022; Hao et al., 2022), the discharge of greenhouse gases such as CH_4_ and N_2_O is also one of the problems that cannot be ignored in the composting of livestock and poultry manure and other nitrogen-rich organic waste. The loss of nitrogen not only decreases the quality of compost, but is also a source of environmental pollution. Thus, research on additives that could reduce greenhouse gas emissions is critical. In addition, the carbon loss caused by the discharge of greenhouse gases (CO_2_, and CH_4_) can also reduce compost-based fertilizer efficiency to varying degrees (Lee et al., 2009; Wang and Zeng, 2018; Cui et al., 2019). Extensive research on the effect of biochar, a commonly used additive, on aerobic composting has been conducted globally (Li et al., 2015). Adding calcium superphosphate to compost can both increase compost nutrients and reduce nitrogen loss. In addition, leavening agents, the most widely used type of conditioner, help to improve the pile body internal conditions and increase material porosity, thus promoting material contact with oxygen to heat the pile body rapidly. At the same, time, leavening agents adjust the initial C/N ratio to balance the nutrient content of compost piles, promote the growth and metabolism of microbes, and thus ultimately improve the quality of the compost products (Barrington et al., 2002). In addition, composting commonly uses leavening agents (including sawdust, crop straw, rice husk, paper sludge, etc.) to adjust the C/N ratio and levels of nutrients (Li X. et al., 2008; Yang et al., 2013; Wang et al., 2016). However, much of the relevant research was conducted under different conditions. For aerobic composting of agricultural solid waste in piles, it is still necessary to systematically study the effects of additives and bacterial agents on the composting process and fertilizer quality, including the changed indexes in the composting process (such as temperature, EC, pH, and NH_4_^+^-N, etc.) and matured of compost (such as C/N, CEC, humus content, and FTIR, etc.). Pile composting could improve the application of aerobic composting on organic solid wastes and expand the practical applications of microbial inoculant.

This research examined the effects of compositing additives and microbial inoculant on the method of on-site stacking at a site in Yangxin County, Binzhou City, Shandong Province. The effects of microbial inoculant and additives on the aerobic composting process with cow dung as the main material were explored to obtain the optimal composting conditions. This research was undertaken to provide a scientific basis for the utilization of organic solid waste processed by on-site stacking. We hope this research can provide a reference for researcher, teachers, students and practitioners for the pile composting of organic solid wastes.

## 2. Materials and methods

### 2.1. Materials

The raw materials were prepared by Shandong Binzhou Jingyang Bio-fertilizer Co., Ltd., Binzhou, China. Cow manure, rice husk, sugar residue, and sheep manure were obtained from farmers around Yangxin County, Binzhou, China. The recovery materials were selected from the composting plant. The microbial inoculant were obtained from the Shandong Academy of Agricultural Sciences, Jinan, China, which had independently developed a combination of strains (composed of *Bacillus subtilis* strain MSJ-2, *Bacillus amyloliquefaciens* strain DB-12, *Bacillus licheniformis* strain DB-18, *Pseudomonas stutzeri* strain ss-1, *Yeast in the ellipse* strain Yj-5, and *Aspergillus oryzae* strain MQ1) isolated from cow dung compost, litter compost, and fruit peel fermentation. Biochar was obtained from Lize Environmental Protection Technology Co., Ltd, Henan Province, China. The biochar was pyrolyzed with corn straw at 500^°^C for 3 h. Superphosphates were obtained from an agricultural supply market in Yangxin County, Zhaiwang Town, Shandong Province, China. The initial physical and chemical properties of compost materials are summarized in Table 1.

**TABLE 1:** The physicochemical properties of raw materials.

Parameters	Cow manure	Sheep manure	Mushroom pomace	Recovery materials	Rice husk	Sugar residue	Calcium superphosphate	Biochar
Moisture content (%)	62.65	64.51	12.60	48.04	10.00	28.44	3.10	2.14
pH	8.98	7.50	7.20	8.91	6.53	3.91	2.34	9.04
EC	5.43	5.22	5.49	6.09	3.31	4.46	6.75	5.85
TC%	31.79	36.21	50.62	14.83	36.00	52.44	0	–
TN%	1.73	2.18	0.97	0.96	0.48	1.65	0	–

### 2.2. Experimental design

The individual Groups were described: Group 1 as contrast, Group 2–4 added microbial inoculant, calcium superphosphate and calcium superphosphate + biochar, respectively. Groups 5–7 had the same microbial inoculant and cow manure, the different substrates (rice husk, sugar residue, and tomato straw) were added to adjust the C/N ratio at 28:1. The stacking experiment was conducted at the indoor facilities of Jingyang Biological Fertilizer Co., Ltd. Zhaiwang Town Industrial Park, Yangxin County, Binzhou City, Shandong Province, China, and the effective volume of each stack was 1 m^3^. Fresh cow dung and various materials were mixed and piled according to the ratios in Table 2. In the first 30 days of composting, the stacks were turned every 5 days to ensure adequate ventilation; from the 31st day to the 45th day, they were turned every 7 days. The aerobic composting lasted for 45 days (from October 1, 2021 to November 14, 2021). The temperature of the upper, middle, and lower layers of the compost was measured three times each day, and the daily average was recorded. Samples were collected at the start of the experiment and on days 10, 18, 26, 34, and 45. The stacks were fully stirred before sampling to make the samples representative. The mass of the sample collected each time was about 800 g, which was randomly divided into two parts. One was stored in a refrigerator at 4^°^C measured directly as fresh samples; after being naturally air-dried, crushed, and passed through a 60-mesh sieve, while the other was used for the determination of other indicators.

**TABLE 2:** Experimental design (quality of dry basis, kg).

Groups	Substrates							Calcium superphosphate (1.25%)	Biochar (1.5%)	Microbial inoculant (5%)
	**Cow manure**	**Sheep manure**	**Mushroom pomace**	**Recovery** **materials**	**Rice husk**	**Sugar residue**	**Tomato straw**			
1	500	200	200	100	–	–	–	–	–	–
2	500	200	200	100	–	–	–	–	–	5
3	500	200	200	100	–	–	–	12.5	–	5
4	500	200	200	100	–	–	–	12.5	15	5
5	750	–	–	–	250	–	–	12.5	15	5
6	450	–	–	–	–	550	–	12.5	15	5
7	628	–	–	–	–	–	372	12.5	15	5

All of the volume of stacking was about 1 m^3^. The qualities of microbial inoculum, calcium superphosphate, and biochar were calculated based on the percentage of total solid mass, while the initial moisture content wasn’t adjust.

### 2.3. Determination indicators and assay methods

Fresh samples were measured for pH, electrical conductivity (EC), the germination index (GI), and ammonium nitrogen. The fresh compost samples were mixed with Milli-Q water at a solid-to-liquid ratio of 1:10, and the relevant indicators were measured after the mixture was allowed to stand for 30 min. EC and pH were determined by the electrode method with a pH/EC meter (Shanghai Leici, Shanghai, China). GI values were determined according to the method in the standard *Organic Fertilizer* (NY 525-2012). Ammonium nitrogen was detected by the indophenol blue colorimetric method.

The air-dried samples were assayed for organic matter (OM), total carbon, total nitrogen, total solids (TS), volatile solids (VS), and humus. Among them, total carbon and total nitrogen were assayed with an automatic elemental analyzer. Humus and cation exchange capacity (CEC) were determined according to the national standard *Determination of Humus Content in Soil* (NY/T 1867-2010). To detect the content of TS and VS, the air-dried and pulverized samples were burned in a muffle furnace at 550^°^C for 4 h and then weighed for determination. OM was measured using the quality difference method, that is, the dry compost samples were burned in a muffle furnace to determine the quality difference. Samples were prepared by the potassium bromide (KBr) pressing method and assayed by Fourier transform infrared spectrometry (FTIR) with a scanning wavelength range of 4,000–400 cm^–1^ and a resolution of 4 cm^–1^. The scanning signals were accumulated 30 times, and the spectral data from different samples were recorded under the same test conditions.

### 2.4. Data processing and analytical methods

All indicators in this experiment were assayed three times to obtain their average values, which are expressed in the form of an average ± standard error. Data were statistically analyzed and plotted using Excel 2018 (Microsoft Corp., Redmond, WA, USA) and Origin Pro 8.0 (OriginLab, Northampton, MA, USA). Parameters were calculated using Excel 2003 (Microsoft Corp.). All figures in this study were analyzed and plotted using Origin 8.0 (OriginLab).

## 3. Results and discussions

### 3.1. Changes in physicochemical properties during composting

#### 3.1.1. Temperature and moisture content

##### 3.1.1.1. Temperature

**FIGURE 1:**
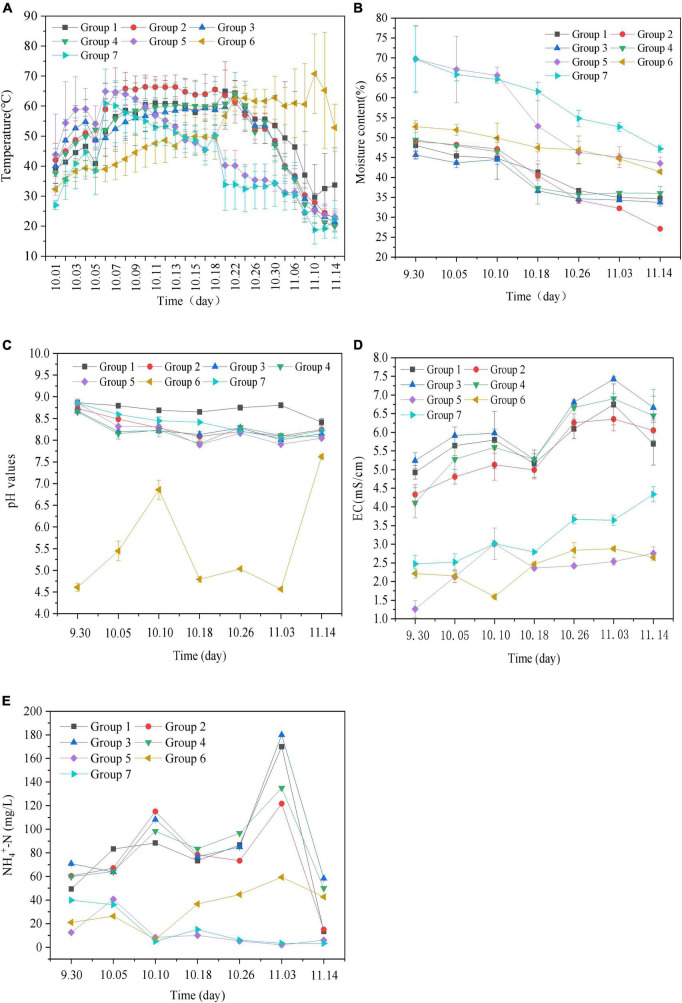
Changes of physicochemical properties during the aerobic composting progress [**(A)** temperature; **(B)** moisture content; **(C)** pH values; **(D)** electrical conductivity (EC) values; **(E)** NH_4_^+^-N values]. Group 1: cow manure + additives, contrast; Group 2: cow manure + additives + microbial inoculant; Group 3: cow manure + additives + microbial inoculant + calcium superphosphate; Group 4: cow manure + additives + microbial inoculant + calcium superphosphate + biochar; Group 5: cow manure + rice husk + microbial inoculant + calcium superphosphate + biochar; Group 6: cow manure + sugar residue + microbial inoculant + calcium superphosphate + biochar; Group 7: cow manure + tomato straw + microbial inoculant + calcium superphosphate + biochar.

Temperature is one of the indicators that reflects whether composting is proceeding smoothly (Atchley and Clark, 1979). During the composting process, changes in temperature directly affect the activity of microorganisms and determine the stability of organic matter (Barthod et al., 2018; Zhang et al., 2018). The effects of microbial inoculant and different additives on temperature during the composting process are summarized in Figure 1A. As can be seen, the temperature of all stacks increased from day 2, reaching 40–60^°^C on day 3; it was highest (70^°^C) on day 8, and the high-temperature period (>50^°^C) lasted for 10 days. As composting progressed, the organic matter in the material became basically decomposed. After 20 days of composting, the temperature of all the stacks except Group 6 began to drop, and the decomposing process was completed after the temperature reached 25–30^°^C. The compost temperature of each treatment experienced a steady increase in the early stage, a moderately high temperature in the middle stage, and a slow decline in the later stage, corresponding with the typical mesophilic, thermophilic, and decomposing stages.

Both microbial inoculant and additives had effects on the rise of temperature during the composting process. Under the same composting conditions of the same substrates (cow manure, sheep manure, mushroom residue, and recycled material), the influence of microbial inoculant on the pile temperature was the most significant in the process of composting, and the high temperature period of composting was occurred 2 days later and lasted for 10 days. Compared with the microorganisms in the recycled material, the added microbial inoculant were more beneficial to the composting process. The temperature changes of Groups 1, 3, and 4 were similar. The average temperatures at the bottom, middle, and top of the reactor were 43.3, 55.7, and 52.8^°^C, respectively (Supplementary Figure 1). The only additive that had no significant effect on pile temperature was superphosphate, but adding biochar did shorten the heating time of the pile and effectively prolonged the duration of the high temperature period, which is conducive to improving the decomposition rate of organic matter in raw compost (Czekala et al., 2016). The gas permeability of the stack was improved, and the effect of oxygen flow and circulation was improved by adding biochar. The temperature of all the piles was maintained at 40–66^°^C during the period from October 6 to 22 (17 days), which is the optimal temperature range for aerobic microorganisms and is conducive to the composting of materials.

In addition, the C/N ratio of the initial compost material regulated by different materials combined with cow manure was 28:1, which was within the appropriate C/N ratio range for microorganisms (Zhu, 2007). However, the content of organic matter in the material that can be degraded and utilized by microorganisms differs (as shown in Table 1 and Supplementary Figure 2), and the temperature of compost showed different tendencies. Compared with other materials, sugar residue (Group 6) has a higher sugar content, which can be more easily degraded and utilized by microorganisms and readily processed into a large number of organic acids in the short term. The pH of the reactor was low, but with a wide range (as shown in Figure 1C), which indirectly affects the activity of microorganisms and eventually slows the initial temperature rise of the reactor. The high temperature period was from 10.24 to 11.10, a total of 17 days. However, when tomato straw (Group 7) and rice husk (Group 5) were added, the temperature pattern of the pile was similar. The structure of tomato straw was complex and not easily degraded by microorganisms, and the addition amount was large (Table 1). After the rapid temperature rise from October 3 onward, the high temperature period was maintained for the shortest time (October 6–13, a total of 8 days), and then, the temperature began to drop sharply to about 35^°^C and stabilized at 20^°^C at the end of composting. However, the initial temperature of Group 5 increased rapidly, reaching the high temperature stage in 7 days, after which the temperature began to decline. The organic matter content of rice husk was low, and the inorganic content in the substrate was increased after the organic matter that could be degraded was exhausted during the compost heating period; at this time, the nutrients available for microbial growth and reproduction were reduced, which led to the gradual decrease of the pile temperature.

##### 3.1.1.2. Moisture content

In the process of composting, water provides the circulation medium for the transport of nutrients and the degradation of organic matter (Atchley and Clark, 1979). The microorganisms absorb the nutrients dissolved in the water for their metabolism and reproduction, and the water functions as a carrier that spreads microbes around, thus promoting the fermentation and decomposition of materials. The effects of microbial inoculant and materials on water content in the composting process are summarized in Figure 1B. In general, the optimum moisture content of compost should be 50–65%. The initial water content of Groups 1–7 ranged from 45 to 71%. As composting progressed, the moisture content of the piles in each treatment gradually decreased. At the end of composting, the moisture content of most piles was stable at 26–46%, and only the moisture content of Group 2 was as low as 26%. The average moisture content of all piles during the process of composting was about 58%, which meets the moisture requirements of compost. Moreover, the material of the piles was not black, indicating that there were no large-scale anaerobic pockets, indicating that the tossing times are reasonable, and the pile was well-ventilated, which basically meets the growth needs of aerobic microorganisms.

Compared with the results of each Group, the calcium superphosphate, biochar, and microbial inoculant (Groups 1–4) had little influence on the water content of the heap, while the substrates had substantial influence on the water content of the heap. Among the groups, Group 6 was similar to Groups 1–4, and the change in water content was small. The potential reasons for the gradual decrease in water content among all groups are as follows: (1) the heat generated by microbial activities led to water evaporation; (2) turning over the pile provided ventilation that caused water loss from the pile. Compared with other materials, the water holding capacity of rice husk (Group 5) and tomato straw (Group 7) were poor, resulting in high water loss during the process of composting.

#### 3.1.2. pH and EC

##### 3.1.2.1. pH

Changes in pH value can change the cell membrane charge of microorganisms and thus affect the absorption of nutrients by microorganisms (Atchley and Clark, 1979; Bai et al., 2020). Therefore, pH is an important parameter affecting microbial activity and the composting process. A pH value of 7.0–8.5 can microbial effectively promote reaction processes, so this range is conducive to the maturation of compost. The effect of microbial inoculant and different materials on pH in the composting process is shown in Figure 1C. At the beginning of composting, except in Group 6 (pH around 4.6), the pH value of the other groups was in the range of 8.6–9.0, corresponding with the optimal pH value for microbial growth (i.e., 6–9). As composting progressed, the pH value exhibited a relatively gentle trend that gradually decreased and stabilized at 8.0–8.5, while the Group 6 value stabilized at about 7.5.

The high or low initial pH value of the material is related to the nature and relative composition of the material. Except for Group 1 and Group 6, the pH values of Groups 2–5 and 7 decreased from day 5 of composting and tended to be stable as composting progressed, finally stabilizing at 8.0–8.5, thus meeting the requirements of decomposition. The analysis indicated that the pH change was mainly caused by the accumulation of organic acids (such as lactic acid, butyric acid, propionic acid, etc.) formed by the decomposition of organic matter, such as fats and carbohydrates, by microorganisms, and the degradation of organic acids and ammonia or ammonium produced by the decomposition of nitrogenous organic matter, which coexist with ammonia/ammonium in the process of composting. Group 1 was the control, and its pH changed more slowly without the addition of microbial inoculant. However, the pH value of Group 6 was lower than that of the other groups, which was related to the influence of composting time and heap temperature. Group 6 had a smaller addition of cow manure and more sugar residue. Although biochar was added, the initial pH value of sugar residue was low, and the sugar content was high, resulting in the slow temperature rise of the pile (as shown in Figure 1A and Supplementary Figure 2). As composting progressed, the substances in the sugar residue of Group 6 that could be easily degraded by microorganisms were indeed degraded. When the composting progress had concluded, the microorganisms used the substances that could not be degraded, and the pH decreased again. After 45 days of composting, although the pH had risen to about 7.5, it did not reach the pH of optimal compost maturation.

##### 3.1.2.2. EC

As one of the critical factors in composting, EC mainly exists in the form of ions in the compost extract. EC is related to the mineralization rate and soluble salt concentration of compost, which directly affects the normal growth of plants. If its value exceeds a certain level, EC will be toxic to crops after compost fertilizer application. The influence of microbial inoculant and different additives on EC is shown in Figure 1D. In general, the EC value of compost must be less than 4 mS⋅cm^–1^. Falling within the normal growth range of plants, compost in Groups 5–7 basically showed a trend of slowly rising first, then slowly declining, and finally stabilizing, that is, it increased in the warming period, reached its peak (2.5–3.0 mS⋅cm^–1^) during the high temperature period, and slowly decreased in the cooling period until it basically stabilized at 2.5–4.0 mS⋅cm^–1^ at the end of composting. However, in contrast to Groups 5–7, Groups 1–4 showed a large range of changes with similar trends, first increasing from 9.30 to 10.10 mS⋅cm^–1^, then decreasing to 4.9–5.3 mS⋅cm^–1^ by October 18, then increasing again, and gradually becoming stable until finally stabilizing at 5.5–6.6 mS⋅cm^–1^. The analysis suggested that this trend was related to the compositions of the substrate. Groups 1–4 were all composed of cow dung, sheep dung, mushroom residue, and recycled material, among which the inorganic component of mushroom residue was high, which affects the mineralization rate of the whole material and led to the overall high EC value.

**FIGURE 2:**
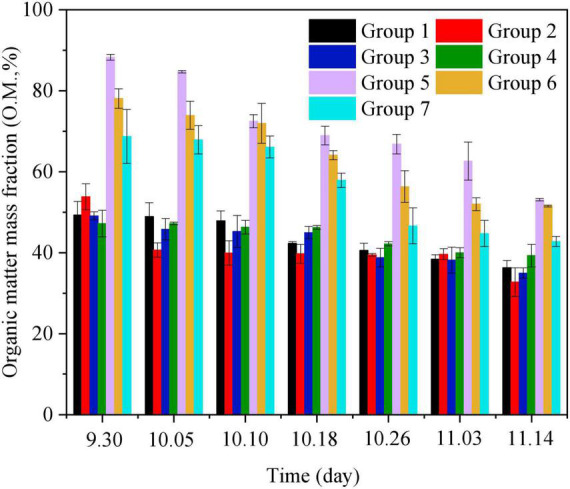
Changes of organic matter mass fraction during the aerobic composting progress. The brief description of the symbols in the figures were similar with Figure 1.

Meanwhile, microorganisms can also degrade soluble salt ions. Compared with Group 1, the EC value of Group 2 decreased by 5.32 ± 0.235% throughout composting. For growth and reproduction, microbes need nitrogen and other nutrient elements (Figure 1E). In the early stage of composting, the EC value gradually increased, which was owing to ammonium, nitrate, and phosphate produced by the decomposition of organic matter, and biochar added was able to adsorb a certain amount of soluble salt ions to reduce the concentration of water-soluble salt. In the late stage of composting, the EC value increased slowly, which may have been owing to the concentration of salt caused by the reduction of water and the degradation of organic matter (Figures 1C, 2). Thus, the growth and reproduction of microorganisms can also reduce the EC value of compost products to some extent.

#### 3.1.3. NH_4_^+^-N

Ammonium content is closely related to the release of ammonia in the composting process, which is the main source of nitrogen loss (Tong et al., 2019; Zainudin et al., 2020). In the processes of composting, nitrogen ammonification, nitrification, denitrification, assimilation, and volatilization reactions produce different nitrogen forms such as ammonium hydroxide, ammonium nitrate, and ammonia, such that the products of composting can be improved. Zucconi et al. (1981) proposed that compost reached maturity when the concentration of NH_4_^+^-N dropped below 400 mg/kg. According to Figure 1E, Group 5 and Group 7 showed a trend of first increasing and then decreasing, while Groups 1–4 had two peaks successively. This may be related to the compositions of the substrate. The nitrogen content of tomato straw (Group 7) and rice husk (Group 5) was low (as shown in Table 1); in addition, the lignin and inorganic components were high, and the components that could be degraded and utilized by microorganisms were low. As composting progressed, the nitrogen content was always in a state of decline, and the NH_4_^+^-N content also decreased. Although the NH_4_^+^-N at the end of composting was less than 400 mg/kg, the composting effect of Group 5 and Group 7 was not optimal according to other composting parameters.

Compared with Group 5 and Group 7, the materials in Groups 1–4 were more easily degraded and utilized by microorganisms, and the NH_4_^+^-N values were generally high, with two peaks. In the early stage of composting, the easily degraded organic nitrogen was used by nitrifying bacteria and converted into NH_4_^+^ by mineralization. Under the action of ammonia-oxidizing bacteria and nitrite-oxidizing bacteria, part of nitrogen undergoes nitrification and dehydrogenation and is transformed into NH_2_OH, NO_2_^–^ and the final product NO_3_^–^ in turn (Coskun et al., 2017). The microbial inoculant initially isolated from cow manure as well as microbes in the recovery materials and cow and sheep manure from the substrates may have included ammonia-oxidizing bacteria and nitrite-oxidizing bacteria, which appeared to play a positive role in the transformation of nitrogen. Under high temperature conditions, NH_3_ is released by deprotonation. As composting progresses, substrates can be degraded to produce more NH_4_^+^-N. Thus the nitrogen content of livestock manure was generally higher than that of crop straw, which was considered to be the main reason for the two observed peaks of NH_4_^+^-N in Groups 1–4.

### 3.2. Degradation of organic matter during composting

The degradation of organic matter is a key parameter reflecting the mineralization and transformation rate of organic nutrients in the composting process (Ren et al., 2019). The effects of substrates and microbial inoculant on organic matter content in the process of material composting are summarized in Figure 2. In general, the organic content of the material in aerobic compost should be controlled within the range of 20–80%. In the process of composting, the trend in organic matter content generally proceeds as follows. (1) During the heating period, soluble and easily decomposed organic matter such as saccharides are degraded first, leading to the continuous heating of the heap. (2) At high temperatures, cellulose and protein are decomposed, and the content of organic matter decreases. (3) In the cooling decomposition stage, the residual lignin and other substances are further decomposed, and the content of organic matter is further reduced. Thus, the degradation of organic matter is rapid in the early stage of composting and gradually tends to stabilize throughout composting.

Over the whole process of composting, the organic matter content of Groups 1–7 showed a gradual decline, with a rapid decline in the first 10 days. Organic matter content was sufficient at the beginning, and microorganisms made extensive use of organic matter for their own propagation and growth, while releasing CO_2_. As the composting process progressed, the decomposition of degradable substrates was completed after 36 days of composting, and microorganisms began to decompose the refractory organic materials. Organic matter degradation rates ranged from 39 to 50% across all groups by the end of composting. Among them, the organic matter content of Groups 5–7 was higher than that of Groups 1–4. According to the analysis, a critical factor mediating the reduction of organic matter was mineralization in the process of composting. However, compared with the rice husk and tomato straw of Groups 5–7, there were more substances easily degraded by microorganisms in Groups 1–4, but the initial content was lower; moreover, the degradation rate of organic matter after composting was lower. In contrast to the trends of other groups, the NH_4_^+^-N content of Group 6 decreased first and then increased. Owing to the high soluble sugar content of sugar residue, microorganisms in the warming stage of compost mainly use free polysaccharides and NH_4_^+^-N for growth and propagation, some of which undergo little change during this stage. As the sugar was depleted, cellulose and lignin were decomposed gradually, and the content of organic matter gradually increased.

### 3.3. Evaluation of post-compost degree of decay

Compost maturity degree is the degree of organic fertilizer humification, which can reflect the degree of microbial degradation of organic matter and the degree of organic material degradation in the process of composting (CCQC 2001, California Compost Quality Council). At present, the evaluation indexes of organic fertilizer maturity include physical indexes (such as temperature, odor, color, etc.), chemical indexes (such as C/N, CEC, and humus content), biological indexes (enzyme activity and microbial community structure changes), and other indexes (such as FTIR and three-dimensional fluorescence spectrum). This study analyzed six quantitative indicators: humus content, CEC, C/N, T, GI, and FTIR.

#### 3.3.1. Humic acid contents

**FIGURE 3:**
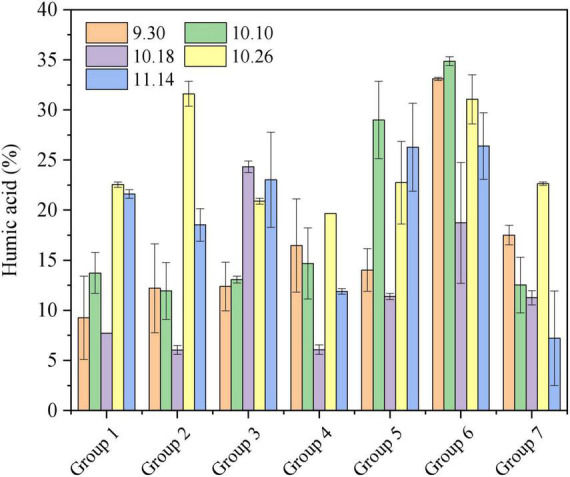
Changes of humic acid during the aerobic composting progress. The brief description of the symbols in the figures were similar with Figure 1.

Compost quality greatly depends on humification (Ma et al., 2022). The change of humus during the composting process with different materials and additives is summarized in Figure 3. The microbial inoculant and additives were able to promote the formation of humus. In the early stage of composting, microorganisms use degradable materials for growth and reproduction, and the temperature rises rapidly. By the middle of composting, the organic matter in the material decomposes rapidly to form humus owing to the continuous action of microorganisms. As the pile body temperature gradually decreases to room temperature, temperatures favorable to microbial population growth return, and microbes become active again and further decompose the refractory organics and simple small molecule organic matter into more stable complex macromolecular organic matter. However, organic matter and humus content showed a trend of declining after rising first.

Substrate type had a significant effect on the humus after composting, and compared with Groups 1–4, and Group 7, the humic acid content of Groups 5–6 was higher than that of humus. The organic matter in rice husk and sugar residue was more easily degraded and utilized by microorganisms. However, the structure of tomato straw made it difficult for microbes to degrade and utilize, resulting in its low humus content after composting. Owing to the nitrogen content of cow manure and sheep manure in the Group 1–4 materials being generally higher than those of Groups 5–7, the materials in Groups 1–4 were easily degraded and utilized by microorganisms, so the humus content was higher in the early and middle stages of composting. In contrast, the degradable components in the recycled materials that formed a portion of Group 1–4 materials were low, resulting in generally low humus content after composting.

Compared with Group 1, microbial inoculant also had a significant effect on the humus after composting (Group 2). Biochar played positive roles on the effect of microbes in the composting process in the following ways: (1) as a place for microbial growth and reproduction; (2) in accelerating the degradation and decay of organic matter that can be used as nutrients for microbes. Thus, the content of nitrate nitrogen and total nitrogen in compost increased, the degradation and decay of organic matter were promoted, and the maturity of compost was improved.

#### 3.3.2. CEC

**FIGURE 4:**
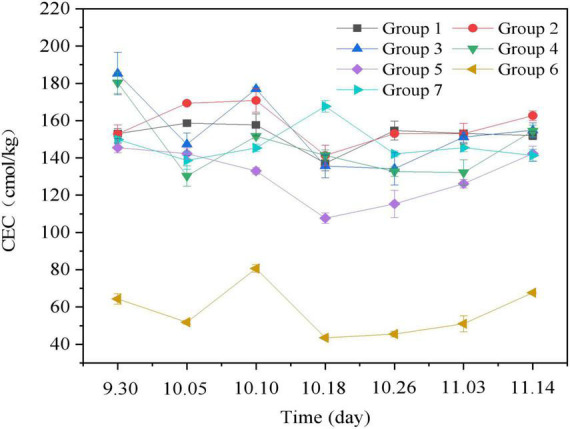
Changes of cation exchange capacity (CEC) during the aerobic composting progress. The brief description of the symbols in the figures were similar with Figure 1.

Cation exchange capacity is an important indicator of the formation and maturation of organic matter in the composting process. The influence of different additives on CEC in the composting process is summarized in Figure 4. When CEC >67 cmol/kg, compost reached the maturity standard (Jiménez and García, 1992). In our experiment, CEC first decreased, then increased, and finally stabilized. After composting, CEC values of Groups 1–7 were 152.00, 162.67, 154.83, 154.50, 142.33, 67.67, and 141.33, respectively. Although the CEC value of Group 6 was greater than 60 cmol/kg, the CEC value throughout the whole composting process was low. The analysis suggests that the content of sugar residue in the Group 6 was high, and the content of degradable substances in the sugar residue was high, which is related to the poor nutrient retention in the material. Owing to the wide range of CEC variation of different materials, compost maturation should be determined using other indicators.

#### 3.3.3. C/N and T

**FIGURE 5:**
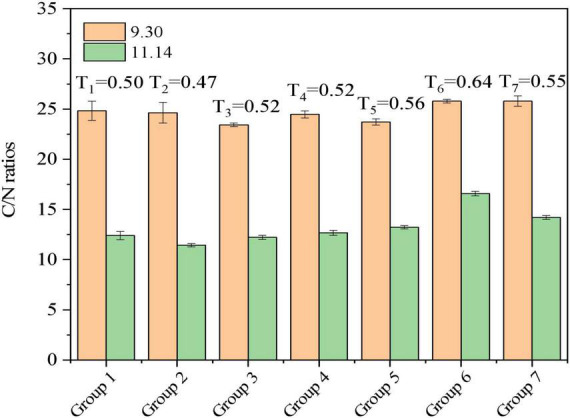
Changes of C/N ratio during the aerobic composting progress. The brief description of the symbols in the figures were similar with Figure 1.

In view of the nutrient requirements of microorganisms, C/N and its change can indirectly reflect the process of obtaining energy and synthesizing new cells through metabolism, and it is also an important index to evaluate the degree of decomposition (Zhu, 2007). Changes in the C/N ratio throughout the aerobic composting process are shown in Figure 5. The initial C/N ratio of general compost materials should be between 25:1 and 30:1, and the initial C/N ratio of Groups 1–7 is within the appropriate range of C/N ratios. When C/N ≤20, it reaches a state of decomposition, and Group 1–7 C/N ratios were between 10:1 and 15:1, which meets the requirements of decomposition. Thus, microorganisms rapidly degraded organic matter and TN in the materials and produced NH_3_ and NH_4_^+^, and the contents of organic matter and TN decreased rapidly. During the high temperature period, a large amount of C is converted into CO_2_ in the physiological and metabolic pathways of microorganisms, and the remaining carbon is converted into storage materials and used for synthesis of protoplasm. In addition, inorganic nitrogen is volatilized and released in the form of NH_3_. At the late stage of composting, the degradation rate of organic matter slowed down, the accumulation of TN decreased, and the C/N ratio further decreased.

As one of the reference indexes for the evaluation of compost maturity, the *T* value represents the C/N ratio after composting to the C/N ratio before composting (Hirai et al., 1983). Generally, when the *T* value is less than or equal to 0.6, compost is well-matured. The results of this study showed that, compared with the control group (Group 1, *T* = 0.50), the *T* values of each Group with an addition of superphosphate (Group 2) of biochar (Group 3) were 0.47 and 0.52, respectively, under the same stacking conditions, indicating their improved decomposition. However, compared with Groups 1, although the *T* values achieved with additions of rice husk (Group 5, *T* = 0.56), sugar residue (Group 6, *T* = 0.64) and tomato straw (Group 7, *T* = 0.55) were higher than that of the control group (Group 1, *T* = 0.50), the *T* value after composting was less than 0.6, except Group 6. This indicates that adding sugar residue material to the pile was not conducive to the deterioration of the materials.

#### 3.3.4. GI

Germination index (GI) values greater than 80% indicated that the compost was fully decomposed and should have no toxic effects on plants (Zucconi et al., 1981). At present, GI is the most effective index to evaluate compost maturation and has been widely used in the study of compost maturation characteristics (Luo et al., 2018). The effects of microbial inoculant and different additives on GI and germination percentage of fertilizer are summarized in Table 3. The germination rates of all groups were in the range of 65–90%, and the average GI was in the range of 1.07–2.10%. In particular, microbial inoculant had a positive effect on the GI value. Except for Group 2 and Group 5, all groups met the GI criteria determining the toxicity of compost to plants (GI > 80%), indicating that the composts of all treatment groups had matured and reached an acceptable degree of basic non-toxicity.

**TABLE 3:** Effects of additives and bacteria on germination index (GI) and germination rate of fertilizer.

Groups	Germination rate	Germination index of seeds
Group 1	88%	1.60
Group 2	65%	1.07
Group 3	80%	1.46
Group 4	90%	1.97
Group 5	75%	1.55
Group 6	88%	2.10
Group 7	85%	1.84

According to EC data (Figure 1D), although EC in all groups was less than 7 mS/cm, soluble salt content did not inhibit germination of wheat seeds. However, as shown in Table 3, compared with the Group 1, the average germination rate of seeds in Group 4 after the addition of biochar was higher, which was consistent with the addition of biochar promoting the decomposition and detoxification of compost. However, the addition of superphosphate had no obvious effect on the seed germination rate. Thus, both superphosphate and biochar helped control nitrogen volatilization, but biochar was more effective. The accumulated substances contained in compost materials, such as NH_3_, volatile fatty acids, and phenols, can inhibit the growth of wheat seeds to some extent, and the addition of biochar can effectively adsorb these substances. The effects of different materials on GI were not significant, which is related to the physical and chemical properties of materials.

#### 3.3.5. FTIR

**FIGURE 6:**
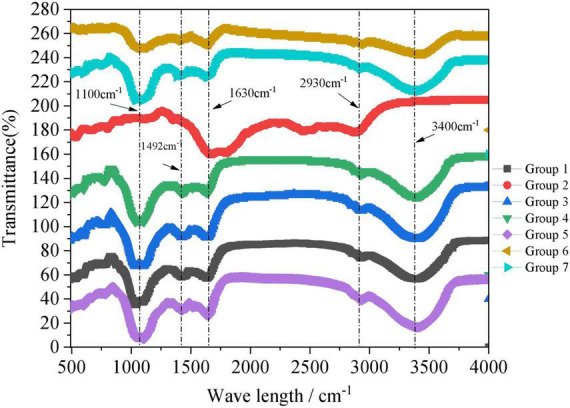
Changes of fourier transform infrared spectrometry (FTIR) during the aerobic composting progress. The brief description of the symbols in the figures were similar with Figure 1.

Through FTIR spectrometry, changes in organic compounds and the degree of decomposition in the process of composting can be understood from a microscopic perspective. Through this technique, the functional group information of relevant organic materials in the process of composting is provided, and the transformation of substances in the process of composting can thus be analyzed, so as to provide a reference for the detection of the degree of decomposition. The effects of microbial inoculant and additives on fertilizer FTIR are shown in Figure 6. There were two strong absorption zones in the composted material (1,100 and 3,400 cm^–1^) and three weak absorbing regions (1,420, 1,620, and 2,930 cm^–1^). Based on previous research (Chefetz et al., 1996; Baddi et al., 2004; Hu et al., 2011; Li et al., 2020), the characteristic peaks in the spectrogram corresponded to the following compounds. Absorption at 3,430–3,410 cm^–1^ indicated the N-H stretching vibration in carbohydrate, protein, and amide compounds. Absorption at 2,930 and 2,850 cm^–1^ indicated the C-H symmetric or antisymmetric stretching vibrations of aliphatic series (-CH_3_ and CH_2_). Absorption at 1,650–1,630 cm^–1^ indicated the COO- antisymmetric stretching vibration of organic acids or the C-O stretching vibration of aromatic rings in lignin. Absorption at 1,492 and 1,425 cm^–1^ indicated inorganic NH_4_^+^, NO_3_, and organic carboxylate COO-. Absorption at 1,170–950 cm^–1^ indicated polysaccharides and was caused by starch cellulose material absorption. The existence of each characteristic peak in Figure 6 indicated that the substrates mainly contained carbohydrates (cellulose, hemicellulose, lignin), proteins, and amide compounds, among other components. Except Group 2, all groups had an obvious absorption peak at 1,100 and 3,400 cm^–1^ in the multi-repeat band, which indicated that the contents of carbohydrates and polysaccharide in the pile were relatively low. In addition, Group 2 has one absorption zone in the composted material (2,930 cm^–1^) as well as one weak absorbing regions (1,492 cm^–1^), which indicates its substrates had more aliphatic group compounds and little lignocellulose. Thus, the added microbial inoculant can promote the efficient degradation of substrates and ultimately yield better compost.

## 4. Conclusion

This study examined the process of aerobic composting with cow manure as the main substrate, with various material compositions and the presence and absence of microbial inoculant and other additives as experimental factors. The effects of microbial inoculant and additives on the dynamic changes of the physical and chemical properties of the compost as well as the degradation, transformation, and decomposition of organic matter in the process of composting were explored. The microbial inoculant and additives all played positive roles in pile composting, and the experimental groups outperformed the control group without any additives. In addition, the microbial inoculant promoted the rapid heating of the pile body relative to composting with the recovery materials alone, and the effects of the microbial inoculant on aerobic composting were related to the organic matter of the substrates and biochar. After composting, all the resulting composts satisfied the conditions of full decomposition. Future research should verify the effects of these fertilizers on saline soil physical and chemical properties, microbial community changes, and plant growth.

## Data availability statement

The original contributions presented in this study are included in the article/Supplementary material, further inquiries can be directed to the corresponding author.

## Author contributions

QY and XL conceived and designed the experiments and analyzed the data. KR and JL performed the experiments. QY wrote the manuscript. SZ wrote the manuscript review and editing. All authors contributed to the article and approved the submitted version.
