# Supplementary material for: Effects of microbial inoculant and additives on pile composting of cow manure
Source: Front Microbiol. 2023 Jan 5;13:1084171. doi: 10.3389/fmicb.2022.1084171 (PMC9850233; doi:10.3389/fmicb.2022.1084171)
Supplement: Supplementary file 1 [file Data_Sheet_1.doc]

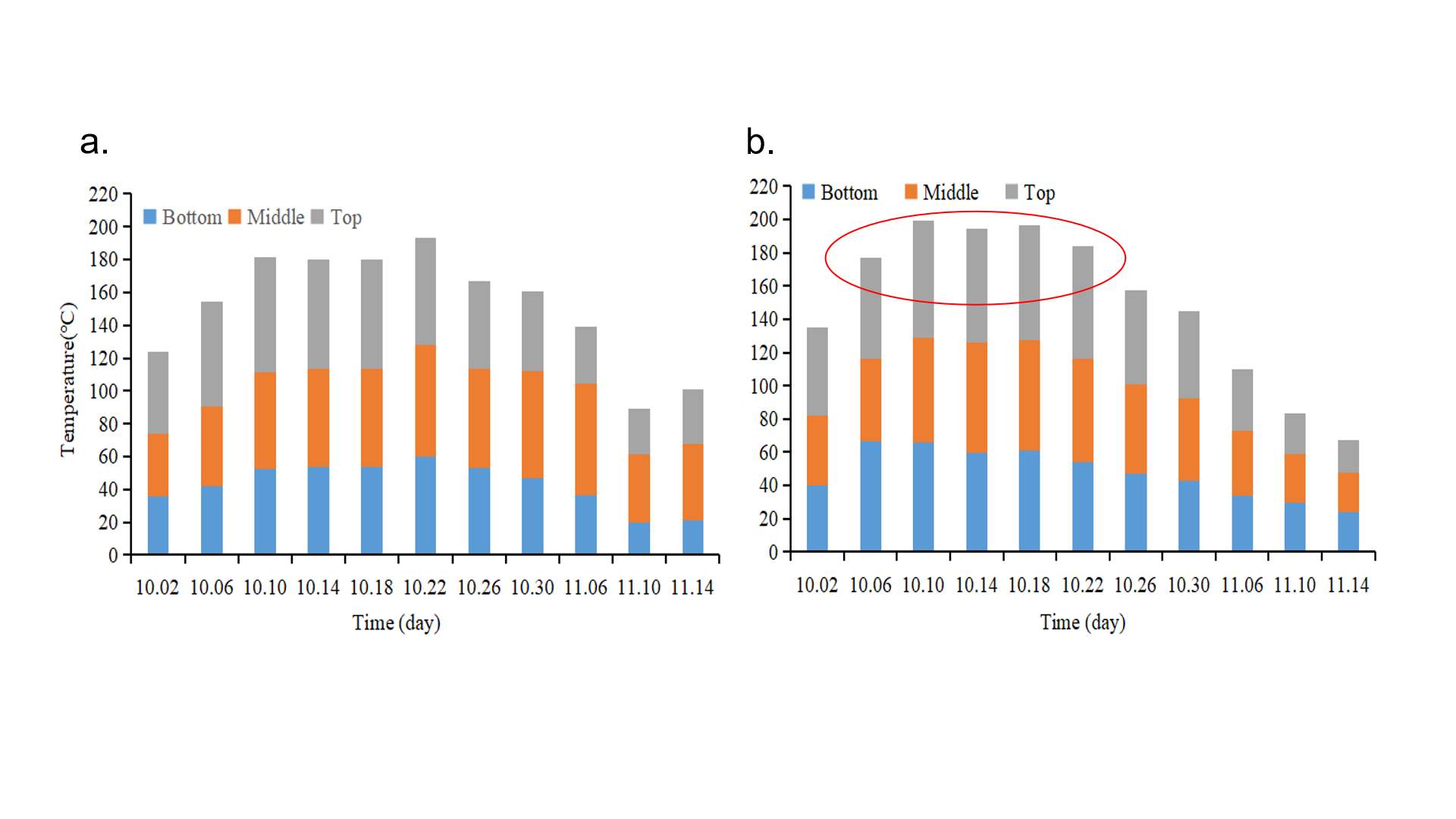


Fig.1 The effect of microbial agent on temperature changes at different pile body position (a. contrast; b. group 1).


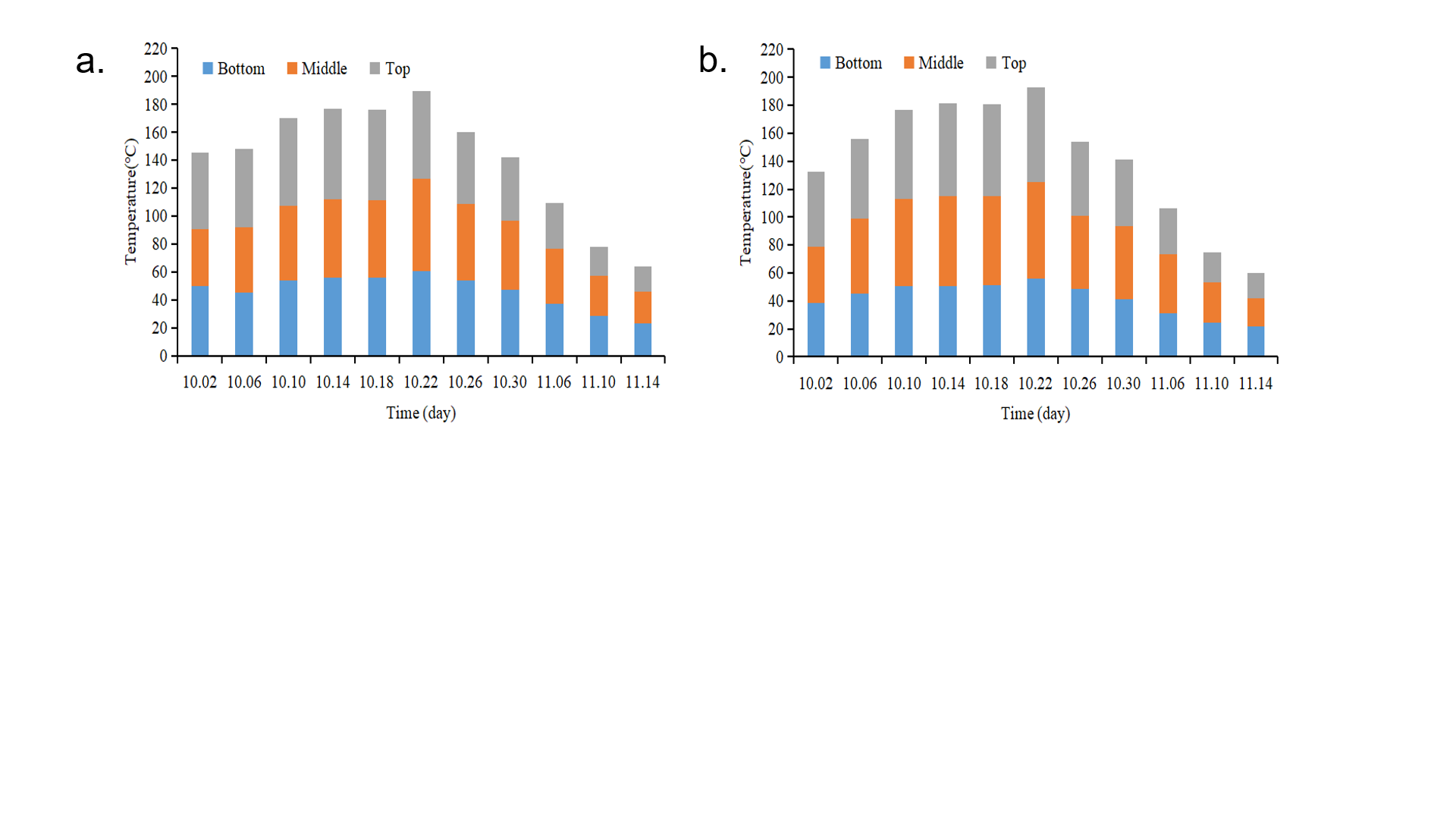


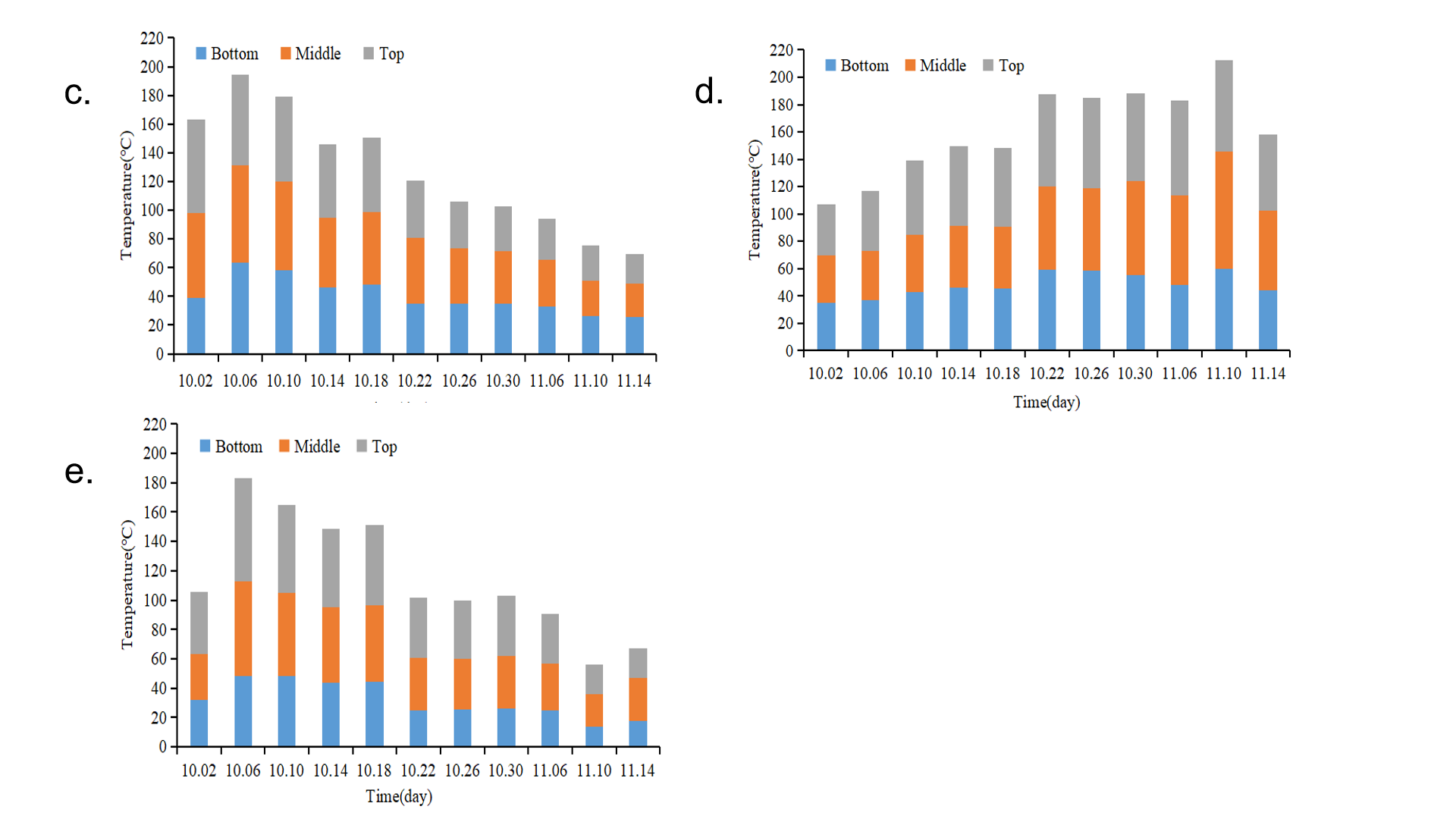


Fig.2 The effect of additives on temperature changes at different pile body position (a. group 3; b. group 4; c. group 5; d. group 6; e. group 7).
